# Harmony but Not Uniformity: Role of Strigolactone in Plants

**DOI:** 10.3390/biom11111616

**Published:** 2021-11-01

**Authors:** Naveed Ur Rehman, Xi Li, Peichun Zeng, Shaoying Guo, Saad Jan, Yunfeng Liu, Yifeng Huang, Qingjun Xie

**Affiliations:** 1 State Key Laboratory for Conservation and Utilization of Subtropical Agro-Bioresources, Guangdong Provincial Key Laboratory of Plant Molecular Breeding, South China Agricultural University, Guangzhou 510642, China; naveed.urrehman@yahoo.com (N.U.R.); 18826231837@163.com (X.L.); zpc2019@stu.scau.edu.cn (P.Z.); syguo6688@163.com (S.G.); 2 Guangdong Laboratory of Lingnan Modern Agriculture, Guangzhou 510642, China; 3 Agriculture Department, Entomology Section Bacha Khan University, Charsadda 24420, Pakistan; drsaadjan@bkuc.edu.pk; 4 State Key Laboratory for Conservation and Utilization of Subtropical Agro-Bioresources, College of Life Sciences and Technology, Guangxi University, Nanning 530004, China; yunfengliu_bio@126.com; 5 Institute of Crop and Nuclear Technology Utilization, Zhejiang Academy of Agricultural Science, Hangzhou 310001, China

**Keywords:** strigolactones, hormones, biosynthesis, signaling and transport

## Abstract

Strigolactones (SLs) represent an important new plant hormone class marked by their multifunctional roles in plants and rhizosphere interactions, which stimulate hyphal branching in arbuscular mycorrhizal fungi (AMF) and seed germination of root parasitic plants. SLs have been broadly implicated in regulating root growth, shoot architecture, leaf senescence, nodulation, and legume–symbionts interaction, as well as a response to various external stimuli, such as abiotic and biotic stresses. These functional properties of SLs enable the genetic engineering of crop plants to improve crop yield and productivity. In this review, the conservation and divergence of SL pathways and its biological processes in multiple plant species have been extensively discussed with a particular emphasis on its interactions with other different phytohormones. These interactions may shed further light on the regulatory networks underlying plant growth, development, and stress responses, ultimately providing certain strategies for promoting crop yield and productivity with the challenges of global climate and environmental changes.

## 1. Introduction

Plant hormones, known as phytohormones, are chemicals produced in very low concentrations to regulate the growth of almost all plant species. The morphological establishment of plant tissue is extensively controlled by phytohormones, including the growth of stems (both upward and sideways), branching, leaf creation, fruit physiology, root growth, life duration, and even natural death. Numerous hormones, including ethylene, abscisic acid (ABA), auxin, cytokinin, and gibberellins (GA), have been widely explored and implicated in the plant developmental processes. Synthetic hormones are frequently applied in several diverse techniques to propagate plants via tissue culture, grafting, cutting, and micropropagation. Strigolactones (SLs) have emerged as a newly defined hormone whose signaling pathways and biosynthesis process have been reported in various plants [1,2,3,4,5]. This newly defined phytohormone was discovered by the research of the parasitic plant *Striga lutea* in the 1960s; the witchweed used the SLs as a signal to initiate germination from the roots of host plants for survival due to the lack of photosynthetic capabilities [6]. Later, a focus has been spotlighted on the SLs as potential targets to create resistance due to a serious infestation caused by parasitic weeds in Africa when using it as a germination stimulant [7,8]. Afterward, SLs are also implicated in serving as a symbiotic signal in arbuscular mycorrhizal fungi (AMF) by the assimilation of inorganic nutrients [9,10]. Notably, the most remarkable effects of SLs are involved in regulating shoot branching of pea, rice, and Arabidopsis [11].

In general, there are two options for studying SLs: genetic characterization of shoot branching mutants and the chemical analysis of SLs. In terms of the former one, SLs are genetically linked with many enhanced shoot branching mutants, including *Ramosus* (*RMS*) of pea (*Pisum sativum*) [12,13,14], *More Axillary Growth* (*MAX*) of Arabidopsis [5,15,16,17], *Decreased Apical Dominance* (*DAD*) of petunia (*Petunia hybrida*) [18,19,20,21], and *Dwarf* or *High*-*Tillering Dwarf* (*D*/*HTD*) of rice (*Oryza sativa*) [2,4,22,23,24,25]. In addition to the shoot branching regulation, SLs also play extra roles in the adaptive response [26,27]. The biosynthesis mechanism of SLs and signal reception and transduction have been elucidated by the characterization of SL-deficient and SL-insensitive mutants [3,5,23,24,28]. SLs are a terpenoid lactone biosynthesized from the carotenoid pathway in plant plastids and are converted to the SL precursor carlactone (CL) via three enzymes: an isomerase DWARF27 (D27) and two carotenoid cleavage dioxygenases, CCD7 and CCD8 [3]. SLs are synthesized in both roots and stems and transported upwards through the xylem to higher parts or extruded to the outside [29,30]. The essential SLs biosynthetic route has been discovered due to the CL discovery in recent years [31,32]. In terms of SLs perception, exploration of a rice SL-insensitive mutant, *dwarf 14* (*d14*), demonstrated that an α/β-fold hydrolase family protein was implicated in SL signaling [2]. In parallel, another smoke-derived butenolide signaling compound, karrikins (KARs), is perceived by D14 homolog, KARRIKIN INSENSITIVE2 (KAI2), which also forms a complex with MAX2 and initiates ubiquitination-dependent degradation of D53 protein homolog, Suppressor of MAX2 1 (SMAX1) [33,34,35,36]. Notably, these KARs are still not fully understood. Although both SLs and KARs signaling pathways share similar features and a close interaction [34,37,38]. Recent studies illustrated that the involvement of SLs/KARs in plant-AMF or -*rhizobia* interactions further expanded their functions beyond their roles in plant development and plant-parasite interactions [14,39,40,41,42], suggesting that SLs or KARs act as a rhizosphere signal to promote the plant-AMF symbioses by promoting spore germination and hyphal branching [33].

Aside from their numerous physiological impacts on root growth, shoot branching, and mycorrhizal branching, SLs have also been linked to legume nodulation [14,39,40,43]. In this review, we intended to highlight the updated progress about the SLs biosynthesis, perception, and signaling, and then further discussed how the SLs crosstalk with other hormones to control the extent of plant development in various plant species.

## 2. SLs Biosynthesis and Signaling Pathway

### 2.1. Biosynthesis Pathway

**Figure 1:**
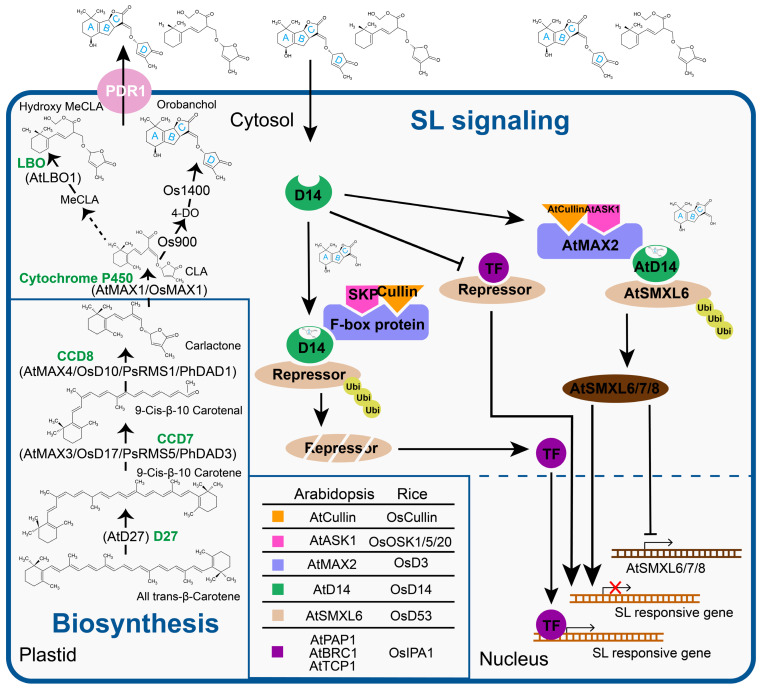
The proposed SL biosynthetic pathway. SLs are derived from the carotenoid pathway, branching out from 9-*cis*-β-10 carotene by the action of iron-binding carotenoid isomerase DWARF27 (*D27*) in rice or *AtD27* to generate 9-*cis*-β-10 carotene. The carotenoid cleavage dioxygenase 7 (*CCD7*), which is encoded by Arabidopsis More Axillary Growth3 (*AtMAX3*), rice *D17*/*HTD1* or pea Ramosus (*RMS*)5, and petunia Decreased Apical Dominance (*DAD*)3, catalyzes 9-*cis*-β-10 carotene into 9-*cis*-β-10 carotenal. *CCD8*, encoded by *AtMAX4/D10/RMS1/DAD1*, then converts 9-*cis*-β-10 carotenal into carlactone. In Arabidopsis, *MAX1* converts carlactone into MeCLA and lateral branching oxidoreductase (LBO) converts the *MAX1*-derived MeCLA (methyl carlactonoate) carlactonoic acid (CLA) that is subsequently converted to methyl carlactonoate (MeCLA) by the unidentified methyl transferase, and finally lateral branching oxidoreductase (LBO) converts the *MAX1*-derived MeCLA into hydroxy methyl carlactonoate (OH-MeCLA), whereas in rice, *MAX1*/*CYP711A2* converts CLA that is then converted to 4-deoxystrigol (4-DO) by CYP711A2 (Os900), and finally CYP711A3 (Os1400) further catalyzes 4-DO to yield orobanchol. Bioactive SLs are perceived by the α/β-fold protein D14 acting as an SL receptor. D14 interacts with *D3*/*AtMAX2* to form the D14-SCF^D3/AtMAX2^ ubiquitination complex, through which the SL signaling repressor D53/SMXL6,7,8 was degraded by a 26S proteosome pathway.

SLs are biosynthesized from the carotenoid metabolism pathway [41], in which the iron (Fe)-containing protein DWARF27 (D27), as well as the carotenoid cleavage dioxygenase 7 (CCD7) and CCD8, are the most important regulators involved in catalyzing SL biosynthesis [11]. These enzymes perform the sequential reactions and finally form the CL as a product [3]. In these reactions, *D27* catalyzes the reversible isomerization of all-trans-β-carotene at C-9 position to produce 9-cis-β-carotene (Figure 1). The *CCD7* cleaves the 9-cis- β-carotene and converts it to the 9-cis-β-apo-10-carotenol, which is utilized by the *CCD8* and then produces the CL (Figure 1). When the *CCD8* is catalyzed by the intramolecular rearrangement of the last portion (D-ring part), the SLs are synthesized, but the chemical reaction mechanism of *CCD8* has yet not been well elucidated [3]. The CL is found as a biosynthetic precursor because it plays a key role in the shoot development of rice mutants (*d27* and *d10*) and also promotes the seeds of *Striga hermonthica* [3]. Recently, the CL has already been identified in plant tissues and also determined its absolute stereochemistry through the liquid chromatography tandem mass spectrometry (LC-MS/MS) technique [32]. Arabidopsis *MAX1* encodes the cytochrome P450 enzyme and its associated homologs convert CL to diverse SLs by unspecified steps [44]. Given the different structures of CL and SLs, it is speculated that there may be additional uncharacterized enzymes to complete the biosynthesis of SL [27], as all SLs reported so far have the same stereochemistry at the C-2 position (R configuration); natural 5-deoxystrigol (5DS) is (+)-5DS or (-)-2′-epi-5DS [45,46]. It was previously reported that carlactonoic acid (CLA) is converted to 5DS and 4DO (4-deoxyorobanchol) in Arabidopsis [31] and is found in both monocotyledonous and dicotyledonous crops [30]. In addition, 5DS lacks oxygen-containing hydroxyl and acetyloxy substituents, and thus forms the simplest form of SLs [47]. Recently, it was observed that the enzyme lateral branching oxidoreductase (LBO) converts the MAX1-derived MeCLA (methyl carlactonoate) into OH-MeCLA (hydroxy methyl carlactonoate) that is significantly involved in the branching of shoot in Arabidopsis [28,32]. Recently, enormous progress has been made in understanding key steps of SL biosynthesis. However, to explore the cell-to-cell transport of SLs, the precursors will expand our understanding of SLs signaling in plant development.

### 2.2. SLs Perception

Different techniques at the genetic level were used to identify the SL receptor genes in petunia, rice, and Arabidopsis, which were termed as *DAD2*, *D14*, and AtD14, respectively [2,5,18,33,38]. The synthetic SLs (GR24) is hydrolyzed by D14 protein into two parts, i.e., D-ring and the inactive ATP-binding cassette (ABC) in vitro. In an in vitro study, SL-like molecules interact with the AtD14 and subsequently hydrolyze [31]. The conserved catalytic triad S-H-D (Ser-His-Asp) mediates the D14 proteins enzymatic activity [5] and is significant for the biological response of D14 proteins as a mutation of D14 in petunia as the Ser residue cannot restore the phenotype of d14 mutant [18].

During the hydrolysis of the SLs, D-OH part makes a complex with the D14 protein and allows for the recruitment of binding partners [38,48,49] (Figure 1). Additionally, there is also a close relationship between the KARRIKIN INSENSITIVE2 (KAI2) clade and the SL receptor D14 clade. However, the biological functions of KAR and SLs are different in regulating plant growth and development, but the *MAX2*-encoding F-box protein is required for both to facilitate their responses [33,50]. The existing evidence implies that SLs are structurally identical signaling molecules sensed by members of the same protein family in plants. Thus, elucidating the ligand-receptor specificity of the D14/KAI2 family is definitely imperative for the field.

### 2.3. Monocots and Dicots Possess a Distinct SL Signal Integration System

The F-box protein and an SKP1-Cullin-F-box (SCF) ubiquitin ligase protein form a complex and are involved in SL signaling [25,51], although the associated regulation mechanism differs between monocot and dicot plants. MAX2 forms the SCF complex in Arabidopsis with AtCullin1 and Arabidopsis serine/threonine kinase 1 (ASK1), whereas the D3 protein interacts with OsCullin1 and Oryza sativa SKP1-LIKE1/5/20 (OSK1/5/20) in rice (Figure 1) [17,23]. *MAX2*/*D3*, similar to other components of the SLs signaling pathway, shows nuclear localization, and their expression patterns are comparable to *D14*/*DAD2* [17,23]. In rice protoplasts, bimolecular fluorescence complementation (BiFC) research confirmed a GR24-mediated interaction between D3 and D14 within the nucleus [23]. The involvement of SL stereoisomers affects the characteristics of this interaction mediated by the SLs-dependent concentration [52]. D3 interacts with at least three different OSKs, implying that MAX2 can interact with several SCF complexes [23].

*D53* and its orthologues *SMXL6*, *SMXL7*, and *SMXL8* in Arabidopsis have been postulated to be transcriptional repressors by interacting with transcriptional factors and transcriptional corepressor proteins TOPLESS (TPL) and TPL-related (TPR) [24,53]. SL molecules can promote the binding of these proteins with the promoter of *D14* receptor genes [53,54]. Simultaneously, SLs can cause the rapid proteasome-mediated degradation of D53 [53], SMXL6 [54], and SMXL7 [36]. SLs degrade the D53 protein, and by repressing its expression in rice, it leads to fewer tillers [53], but the proteasome inhibitor (MG132) cannot affect the abundance of D53 protein [24,53]. *Suppressor of MAX2*-*Like6* to *8* (*SMXL6* to *8*), orthologs of *D53*, function redundantly since the reduced branching phenotype could only be found in the triple mutant *smxl6*/*7*/*8* [54]. SMXL6 can also act as a transcriptional repressor in conjunction with the TPL/TPR proteins to regulate shoot branching, leaf elongation, and anthocyanin production, primarily via suppressing the transcription of *Branched 1* (*BRC1*), *TCP Domain Protein 1* (*TCP1*), and *Production of Anthocyanin Pigment 1* (*PAP1*) [55], respectively.

Both rice and Arabidopsis have SL repressors that contain a highly conserved ethylene-responsive element-binding factor-associated amphiphilic repression (EAR) motif of five amino acids (F/L-D-L-N-L). This motif has been assumed to interact with the transcriptional corepressors TPL and TOPLESS-related Proteins 2 (TPR2) [36,53,56] (Figure 1) [54]. In Arabidopsis, SMXL7, D14, and MAX2 interact in the nucleus in an SL-dependent manner as well [57].

The existence of at least three SL repressors in *A. thaliana* suggests that the SLs signaling pathway is likely regulated in a variety of ways, which expands the spectrum of the effects on plant growth. This hypothesis will be confirmed by studies on individual SMXLs and the discovery of genes controlled by SCF complexes containing distinct repressors. In addition, the perception of SLs, together with the degradation of D53/SMXL6 to 8, involving with the successive degradation of the signal molecule, receptor, and downstream effector, is a unique phenomenon among plant hormones. Identifying additional transcriptional targets downstream of SLs signaling is critical to improving our understanding about SLs’ signaling transduction mechanism.

### 2.4. SL Transport

In petunia, pea, and Arabidopsis, it has already been certified that the SLs are transported from root to shoot at the early stages of grafting assay, but no movement from shoot to root was revealed [20,58,59].

The Pleiotropic Drug Resistance 1 (PDR1) protein is required to export SLs in petunia [60,61] and its expression was found at the tips of the roots, in the HPCs (hypodermal passage cells), root vasculature proximity, and stem nodes (close to the axillary buds). The HPC received specific attention since it is a recognized entry site for mycorrhizal fungi [62]. Interestingly, the *PDR* gene was distributed in patches and identified with HPCs, through which AMF penetrate into the cortex, implying that SLs may function inside as well as outside the roots to establish a symbiosis with AMF [60,61]. PDR1 is a defined strigolactone transporter found apically in root tip cortex cells, the root hypodermis, and laterally in HPCs [60]. It has been reported that the ABC protein is essential in the regulation of outward- and shoot-ward-directed SLs trafficking. The SLs exudation into the soil is consistent and the expression of *PDR1* is high in the root tips accompanied by the highest expression of the SL-biosynthesis gene [63]. Notably, no orthologue of *PDR1* has been found in Arabidopsis, and the closest homologue of *PDR1* is an ABC transporter (*ABCG40*), which presents low identity [64]. In Arabidopsis, mycorrhization is absent due to the absence of a transporter protein compared to the production and signaling components [64]. These findings suggested that additional components rather than *PDR1* may be involved in SLs transport in Arabidopsis [60,61]. However, overexpression of *PDR1* can result in enhanced tolerance to synthetic SLs at high concentrations and increase SL export from Arabidopsis roots [60,61]. So far, certain components involved in SLs biosynthesis, signaling transduction, and transport have been characterized in some plant species (Table 1). Therefore, further identification of the homologous proteins of the given components might extend our understanding of the conservation and divergence of the SLs functions among various plant species.

However, much remains to be learned about SL biosynthesis and signaling pathways. It is likely that SL diversity provides redundancy, causing mutations of a single SL diversification gene to exhibit a mild phenotype or none [28]. Forward genetics as an effective method of identifying the genes is involved, and new tools are likely to be required to fully delineate SL biosynthesis for diverse SLs. Filling this critical gap in SL biology would greatly enhance the potential to modulate SL diversity for sustainable crop production.

## 3. SLs in Controlling the Plant Architecture

### 3.1. SL Signaling Controls the Branching through Nuclear Resided Transcription Machinery

**Figure 2:**
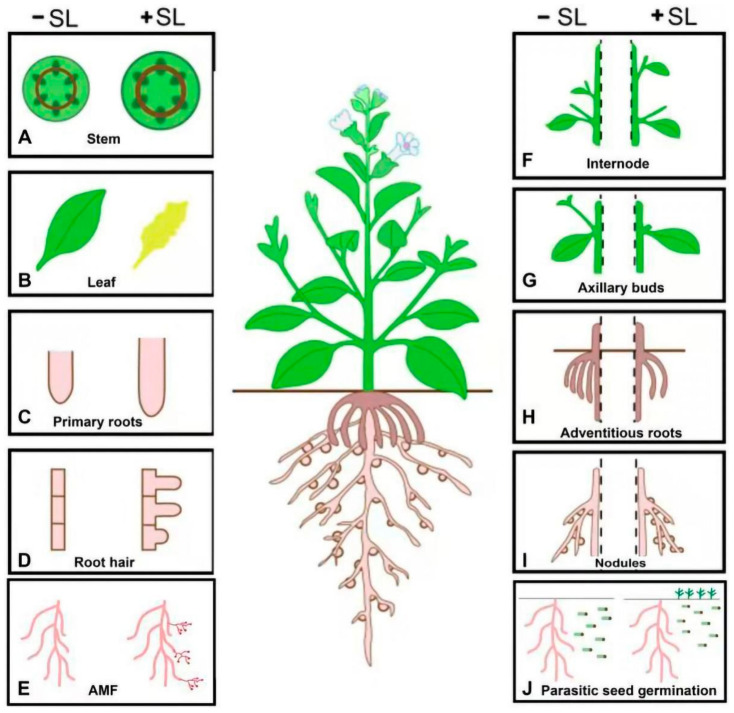
The roles and effects of SLs in plant development. These roles have been confirmed in multiple species and/or with SL-deficient mutants, such as (**A**) SL promotes stem elongation, (**B**) leaf senescence, (**C**) primary root length, (**D**) increases root hairs, (**E**) increase hyphal branching, (**F**) promotes internode length, (**G**) inhibits bud outgrowth, (**H**) inhibits adventitious roots, (**I**) increases nodules numbers, and (**J**) promotes parasitic seed germination.

Suppression of shoot branching is a well-known function of strigolactones (Figure 2). To understand the role of SLs in branching, two mechanisms have been proposed. Studies in rice and pea showed that SLs affect the *OsTB1*/*PsBRC1* TCP transcription factors that suppress the outgrowth of axillary buds [69,70]. *OsTB1*/*PsBRC1* is also a key integrator in numerous other pathways in peas, including the recently identified sucrose signaling system and the cytokinin route [71]. Interestingly, the maize ortholog *TB1* encoding a TCP transcription factor seems to act independently of SLs to repress shoot branching [72]. Moreover, the rice *MADS57* and *IPA1*/*OsSPL14* transcription factors that regulate shoot branching have also been linked to the SL signaling pathway, but it is still debatable how these transcription factors mediate SL signaling and how they lie downstream of the *D14*, *D3*, and *D53* axis [73,74]. Recently, exogenous SLs have been shown to inhibit the outgrowth of axillary buds in apple (*Malus spectabilis*) [75]. The maize mutant *Brc1* developed fewer branches than the SL-deficient mutant [69], suggesting an independent effect of *BRC1* on shoot branching. In Arabidopsis, the rapid elimination of polar auxin efflux carrier was detected in the xylem parenchyma cells of the main stem [76]. This action of SLs is based on auxin transport canalization-dependent processes that increase the competition to export auxin into the mainstream [76,77]. It is still unclear if these processes influence shoots branching or act differently from bud outgrowth phases. All of these investigations show that SLs act as a negative regulator of branching. Shoot branching is a complex process, and it is conceivable that numerous interacting regulatory systems have gathered over the course of its long evolutionary history. Understanding how SL regulation of branching has evolved throughout terrestrial plants could potentially provide major insights into the mechanism of SL action. The generation of extensive mutants by precise gene editing and the application of particular inhibitors at various biosynthetic steps may reveal the types and crosstalk of various shoot branching inhibitory hormones. In comparison to other plant hormones, SLs appears to regulate a few numbers of genes, and thus large-scale transcriptome studies would facilitate the understanding of the possible SLs targets in bud tissue, where SLs is thought to govern bud outgrowth.

### 3.2. Interplay between SLs Signaling and Auxin Flux during Root Development

SLs influence several aspects of root development and root architecture of the whole plant root system. Plants have different types of root systems, such as radical roots that arise from seed directly and develop as a primary root. A complex root system usually initiates from primary roots and then forms lateral roots or secondary roots. Another type of root system is known as adventitious roots, which usually develop from plant parts or other roots such as a stem, leaf, or branch. In monocot, the growth of adventitious roots occurs from the shoot nodes that dominate the entire root system of the plant. Studies indicated that SLs positively regulate the primary root length in Arabidopsis (Figure 2), as well as promote root hairs and inhibits the formation of lateral root and adventitious roots [78]. The exogenous application of synthetic SLs restored the short-rooted phenotypes in SL-deficient mutants but not in SL-insensitive mutants (Figure 2) [79]. The length of the primary root increases by the exogenous application of GR24 due to an increase in the numbers of cells in the transition and meristem zones [79]. A previous study indicated that the size of the meristem and transition zones in roots could not be increased without the gradient of auxin concentration [80]. Therefore, there should be a crosstalk between SLs and auxin in root development. A further study revealed that application of SLs reduced the intensities of the auxin efflux carriers PIN1, PIN3, and PIN7 (GFP) florescence, suggesting that primary root elongation was controlled by SLs through the regulation of auxin flux in Arabidopsis [79]. Distinctly, it has also been stated that the application of SLs (GR24) suppressed the formation of the adventitious root in intact Arabidopsis hypocotyls and the stem cutting of pea [81]. Nevertheless, it is also proposed that the SLs act independently of auxin signaling, whereas the interaction between SLs and auxin seems quite complex. In the auxin signaling mutant *axr1*, the acceleration of adventitious root formation was stopped but this defect was not found in *max* mutants, demonstrating that the suppression of adventitious root formation required the auxin signaling [81]. A large portion of the root system is composed of crown roots or adventitious roots in rice. Unlike Arabidopsis and pea, the rice SLs positively regulate the elongation of crown roots [82]. Furthermore, it was observed that the elongation of crown roots is due to the increase in the cell number [82]. This differential effect may account for different involvement of adventitious roots to the entire root system. Previously, it was observed that SLs and ethylene coordinately enhanced the root hairs through a shared regulatory pathway in Arabidopsis [83]. Furthermore, it was suggested that there is likely a possible junction of auxin and SLs pathways in the regulation of the root hair elongation [83]. In addition, SLs also negatively regulate the formation of lateral roots under normal Pi-sufficient circumstances [79,83]. Taken together, these findings elaborate that SLs may function in different aspects of the root system, but whether the relevant regulatory mechanism is conserved or not among different plants is still poorly understood. SLs are hypothesized to control the length of primary root (PR), the production of lateral root (LR), and adventitious roots (ARs). The interaction with other hormones and the nature of these actions differ among species and growth conditions. Therefore, SL-mediated upregulation of primary root length and root hairs might have applications in improving water and nutrient uptake.

### 3.3. SL Signaling in Plant Developmental Age with Reference to Leaf Senescence

Leaf senescence is typically a natural death process of monocarpic plants, implying that it is the final stage of plant development. Therefore, the timing of leaf senescence is generally controlled by developmental age [84]. During senescence, the breakdown of many cellular proteins, carbohydrates, and lipids were handled by hydrolytic enzymes, and the resulting metabolites were then transported via the phloem back into the main body of the plant for nutrient recycling [84]. The internal cues involved in leaf senescence include various plant hormones. ABA, jasmonic acid, and ethylene can induce the senescence of leaves, while cytokinins are strong inhibitors of leaf senescence [85]. In addition to these phytohormones, SLs also appear to be involved in regulating leaf senescence because a delayed leaf senescence phenotype was found in the SL-deficient and SL-insensitive mutants (Figure 2) [18]. The Arabidopsis mutants *ore9* were observed with more shoot branches with delayed leaf senescence that was identical to the *max2* mutant [51]. Recently, it was observed that a bamboo leaf segment showed response to GR24, confirming SL is a positive regulator in senescence. Interestingly, SL induces cell death in dark conditions rather than in light conditions, and this effect can be blocked by treatment with sugars, such as glucose (Glc) [86]. The mutation of the orthologue of MAX2, the *d3* mutant in rice, also resulted in delaying leaf senescence [87]. Similar to the SL-insensitive mutants, the SLs synthetic mutants also showed delayed leaf senescence in *petunia* and *lotus*. In addition, the phenomenon of increased branching with delayed leaf senescence was also identified in the *ccd7*/*max3* mutant of *Lotus japonicas* [88]. Moreover, the SLs biosynthesis-related genes *MAX1*, *MAX3*, and *MAX4* were observed with increased transcript levels in the senescing leaves, further suggesting a vital role of SLs in leaf senescence (Figure 2) [78]. Recently it was observed that SLs also promote leaf elongation in an EAR-dependent manner through *SMXL6* [55]. However, the leaf shape of the *s678 brc1*-*6* quadruple mutant was similar to that of the *s678* triple mutants, raising the possibility that SLs also control leaf development through other downstream genes [55]. Furthermore, the expression of *TCP1*, which regulates leaf development was repressed more in *max3*-*9* but dramatically increased in *s678* and *max3*-*9 s678* mutants [55], and the transcriptional regulation of *TCP1* depended on the EAR motif of SMXL6 [55].

### 3.4. SLs Elongates the Internode Length

In the SL-deficient and SL-response mutants of rice, pea, tomato, canola, and Arabidopsis, a short stature phenotype was observed in comparison to wild-type plants, suggesting that SLs also promote internode elongation (Figure 2) [12,25,29,51,58,89,90,91]. For example, the rice *htd1*/*d17* mutants deficient in SLs production showed reduced height as compared to wild-type plants [66]. However, the plant height of the *d17* mutant was increased by removing the tillers, but this plant height recovery was not fully restored as compared to that of wild-type plants. Further analysis revealed that the incomplete restoration of the *d17*/*htd1* mutants is due to the diversion of energy from the main stem to the tiller before the removal of tillers, or their SLs deficiency [66]. The exogenous application of GR24 can significantly restore the plant height of SL-deficient mutants of rice [11]. Notably, a recent study illustrated that the SLs affect the level of GA (GA1), and also affect GA signaling in pea; however, the GA and SLs act independently in enhancing the internode elongation in pea [49].

### 3.5. Role of SLs in Nodulation

SLs also have important roles in the regulation of nodules that occur under nitrogen deficiency in legumes [14,39,40,42]. In pea, the autoregulation of nodulation (AON) mutants is epistatic to SLs and brassinosteroid biosynthesis genes. These two hormones, with a role in the promotion of nodulation, probably act independently of the AON system [14]. Furthermore, the nodule number significantly increased by the application of a low concentration of GR24 in alfalfa (Figure 2) [42]. Additionally, SL-deficient mutants of pea had reduced nodule numbers, and the application of synthetic GR24 increased the nodule number [39]. The pea SL-deficient mutant *rms1* has about 40% fewer nodules than the wild type, and the application of GR24 phenotypes partially rescued the pea plant [39]. Likewise, it has been reported that *Lotus japonicus* knockdown plants, with about 80% less reduction in SLs, carried 20% fewer nodules compared with the control plants [88]. The above-mentioned observation through different experiments demonstrated a positive function of SLs in determinate (*L. japonicus*) and indeterminate (pea and alfalfa) nodules development.

However, the pea mutant *rms4* defective in strigolactone signaling carries the maximum number of nodules than the wild type in comparison to rms1 plants [14]. Moreover, transgenic roots of soybean with the over-expression of *GmMAX2a* have a higher number of nodules, while *GmMAX2a*-knockdown roots have fewer numbers of nodules (Figure 2) [33]. Soybean nodules belong to the determinate type, which is different from indeterminate nodules of pea and *M. truncatula*, not only in structure and development but also in other aspects of nodulation. *MAX2* and *RMS4* also participate in the SL/KAR pathway differently depending on the plant ecology [50]. The evolutionary investigation of D14/KAI and MAX2 homologs from leguminous species revealed that these proteins developed into distinct clusters, corresponding to indeterminate and determinate nodules [33]. Furthermore, GR24 develops the root architecture in *M. truncatula* and influences nodulation positively or negatively depending on the hormone levels [92]. In legumes, SLs positively regulate nodulation and thereby play an important role in nitrogen acquisition and this may lead to applications enhancing nutrient access and yield in legumes.

## 4. SLs Are Signals for Plant Interactions

Originally, SLs were recognized as the stimulus for germination of parasitic plants [93]. For hyphal branching in AMF, SLs were also known as stimulants [9,10,93]. Furthermore, SLs were identified to have a positive influence on nodule formation in the interaction process of legume–rhizobium [39].

### 4.1. Involvement of SL in Hyphal Branching Induction during Mycorrhizal Symbiosis

Arbuscular mycorrhizal (AM) symbiosis is the most predominant interaction on the ground, which contains the interaction between the soil AMF and higher plant roots. The AMF formed a symbiotic association with most land plants, in maximum cases donating to the development of the plant, especially under an acceptable growth state [62,94]. Studies revealed that SLs induce hyphal branching in many AMF as they are capable of inducing germination of seeds in parasitic plants. These SL molecules are present at a very low level in the rhizosphere at sub-nanogram [10]. Likewise, the hyphal branching in AMF is efficiently induced by synthetic SLs (GR24) (Figure 2) [10]. Furthermore, the rhizosphere mutants of pea and tomato that are deficient in SL production showed reduced AMF hyphal branches compared to wild-type plants [90]. However, lower colonization rates were observed in the tomato SL-biosynthesis mutant in comparison to the wild type, and the differences were minimized when the plants were inoculated with spores and hyphae or infected roots [90]. Additionally, in the roots of mycorrhizal tomato plants, the production of SLs was reported to be significantly reduced [95]. Furthermore, AMF negatively regulate SLs through a feedback loop, otherwise, the colonization of AMF has a positive influence on the enhancement of AM symbiosis that increases Pi accumulation and induces the suppression of SLs biosynthesis.

### 4.2. SLs Guide Germination of Parasitic Plants

The connection of parasitic weeds *Striga* and *Orobanche* with the plant roots is a known case in which the main parallelism with the AM signaling pathway has been detected [95,96]. The SLs permit the parasitic plant germination from the root surface at the right distance, temperature, moisture, and nutritional levels in the rhizosphere of the host, and at right time in the season. These compounds are found in the host plants root exudates and also occurred in the non-host plants for the germination of *Striga* and *Orobanche* seeds [49,97,98,99,100]. At a very low concentration, SLs are extremely active and endogenous SLs induce germination of *Orobanche* seeds [101]. Studies revealed that naturally occurring SLs are 100 times more active than GR24 and the activity of germination was significantly higher in the *Striga* seed than the GR24 treatment alone by mixing a GR24 solution with root exudates from cowpea plants [100].

### 4.3. SLs Symbiosis with Rhizobium spp.

*Rhizobium* spp., the nitrogen-fixing bacteria, play an important part in the development of nodule and symbiotic interaction with legume roots, such as bean (*Phaseolus vulgaris*), pea, alfalfa (*Medicago sativa*), and clover (*Trifolium* spp.) [102]. So far, SLs are found in all leguminous plants, and play a key regulating role during the rhizobium–legume interaction [47,98]. Currently, it was indicated that the *Orobanche* plant infection could be controlled by the conserved symbiotic pathway that mediates AM and nodule formation. In communication with *Orobanche crenata*, the mutant plants of *M. truncatula* and *Pisum sativum* exhibited different patterns. However, it was observed that SLs are required in determining the optimum nodule numbers but are not important for the development of nodules [14,33,40]. Recently, it was observed that the *Medicago truncutula* mutant *mtabcg59* displayed a reduced level of mycorrhization compared to the wild-type (WT) plants but had no impact on the number of nodules after *Sinorhizobium meliloti* inoculation [103]. The production and secretion of SLs from plant roots can regulate rhizobium bacteria coexistence with legumes, and the coexistence of plants with AMF can improve plant development and growth, and further control the metabolism of the plant under normal and stressful conditions.

## 5. SL Crosstalk with Different Hormones

### 5.1. SL Interaction with Auxin in Controlling Plant Architecture

**Figure 3:**
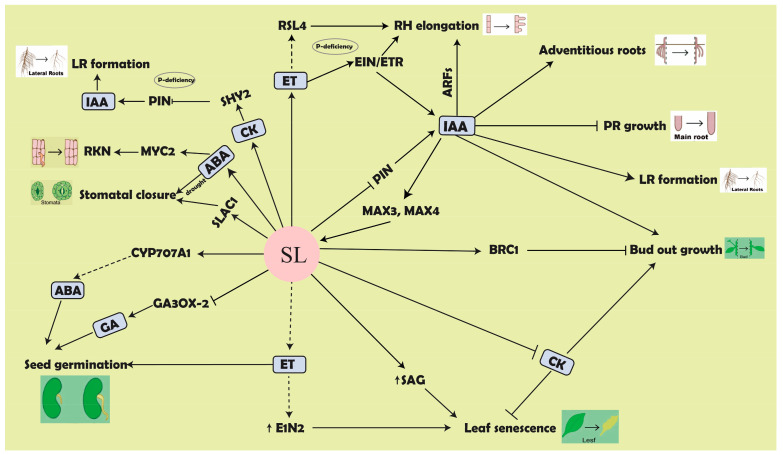
Possible interaction of SLs with different phytohormones. SLs regulate different features by interaction with other phytohormones. SLs increase the PR length and inhibit the LR initiation and bud outgrowth by suppressing the auxin transporter (PIN). SLs increase the abundance of BRC1 and also inhibit CK activity, which as a result stops the bud outgrowth. The expression of SAG increases which stimulates the leaf senescence. The interaction of SLs and ABA is dependent on the condition, such as in stressful conditions both phytohormones up-regulate. SLs regulate the ABA catabolic gene CYP707A1 and decrease the ABA content to regulate seed germination, while under hostile conditions, SLs activate SLAC1 which induce the closure of stomata in an ABA-independent manner. SLs stop the accumulation of ABA and inhibit MYC2 expression and RKN infection. SLs decrease the GA3ox2, a key enzyme in GA production and 9-cis-epoxycarotenoid dioxygenases (NCED), at 32 °C to regulate seed germination. SL increases the ET that increases the EIN and RSL4 signaling and regulates RH and leaf senescence. Abbreviations: PR, primary roots; LR, lateral roots; RH, root hair; IAA, auxin; GA, gibberellic acid; CK, cytokinin; ABA, abscisic acid; ET, ethylene; SL, strigolactone; SAG, senescence-associated gene; SLAC1, Slow Anion Channel Activating 1; RKN, root-knot nematode.

The initiation of bud outgrowth is associated with auxin export from buds, and hence, it is supposed that the export of auxin is needed for the activation of bud outgrowth [104]. Down-regulation of the expression of PIN-formed (PIN) proteins, a family of transporters responsible for auxin influx and efflux from cells, as well as their polarized localization on the plasma membrane [76,105,106], dampens the polar auxin transport (PAT) stream auxin sink strength, hampers auxin export from buds and canalization, and ultimately results in the repression of bud development (Figure 3). Previously, the higher transcript level of *PIN1*, *3*, *4*, and *6* was recorded in *max* mutants of Arabidopsis in which the auxin transport levels were increased in the primary stem compared to the wild type [107]. Moreover, the SLs suppressed the PAT stream in a MAX2-dependent manner due to the exogenous application of GR24 that reduced the auxin transport basipetal and PIN1 accumulation in the parenchyma cells of xylem in the biosynthetic *max* mutants and the wild type but not in the *max2* mutant [105,106]. Recent studies indicated that depending on the auxin transport status, SLs either reduce or increase shoot branching of the treated plants by using different concentrations of GR24 [76,108]. It has been observed that increased transport levels in auxin as a result of decapitation occur slowly in the main stem to clarify the increased number of buds outgrowth. These results further confirmed that shoot branching was promoted by using a low concentration of GR24 (10 nM), while reduced branching by a higher concentration of GR24 (0.1 to 1 μM) in the *tir3* mutant [76].

Similarly, SLs systemically regulate competition among the buds to release auxins into the main stem depending on the auxin transport [76,106,109]. SLs interact with auxin to influence root development in the same way as shoot branching does, by influencing auxin sensitivity [110], PAT from shoot to root [111], and auxin flux within root tissues [112]. Auxin signal modules act downstream of SLs since GR24 has been found to influence PIN protein polarization and localization, as well as LR-forming capacity (Figure 3) [79,112,113]. Furthermore, SLs regulated primary root growth and mediated through the inhibition of PIN1 [68,90]. Notably, the negative effect of SLs on PIN1 in the root is much more sever and more specific compared to the shoot, while no obvious effect of GR24 on PIN1 accumulation was observed in the root apex [76]. However, the effects of SLs on PINs are only expected to happened specifically in the endodermis cells of the transition zone (TZ), however, visualization of this process is technically challenging. Furthermore, four hours of GR24 treatment reduced the expression of the auxin-biosynthesis genes *YUC3* and *YUC5* [55]. In contrast, auxin boosts the expression of the SLs-biosynthesis genes *MAX3* and *MAX4* (Figure 3) [65,114,115]. In addition, it was observed that both endogenous and exogenous SLs interact with canalization-dependent developmental processes, namely auxin feedback on PIN polarity and clathrin-mediated endocytosis of PIN proteins in pea and Arabidopsis [116]. Similarly, *GH3* is an early auxin-responsive gene, and apple (*Malus domestica*) *MdGH3*-*2* and *MdGH3*-*12*, was shown to be significantly induced by AM fungi. It was revealed that down-regulating the expression of these genes by RNAi decreases the expression of SLs-biosynthesis genes, indicating that *GH3* expression has a positive effect on the SLs biosynthesis [117]; however, further research is needed to understand the SLs and auxin conjugation by GH3. The modulation of auxin levels and gradients via their influence on PINs is a common target for many plant hormones. However, the actual process by which SLs accomplish this remains a mystery. This is complicated because multiple hormonal and environmental cues interact with one another.

### 5.2. SL-Mediated Response in Plants Involving ABA

Several phytohormones have been observed to control the adaptive response to environmental stresses together, such as ABA, brassinosteroids, and cytokinin (CK) [118]. The association between the SLs and ABA metabolism has been observed due to their involvement in carotenoid cleavage enzymes and shared carotenoid precursors [119]. However, up to now, only limited experiments have been performed to find out these interactions, with inconsistent results to some extent [120]. It has been widely reported that the ABA and SLs are involved in the regulation of a wide range of physiological mechanisms [121]. A positive association between SLs biosynthesis and ABA was confirmed by the specific inhibitor application for ABA-insensitive mutants and 9-cis-epoxycarotenoid dioxygenases (NCED) enzymes in the shoots with a set of tomato mutants that block at several steps in ABA synthesis [95]. A decreased ABA level was recorded in the SL-deficient tomato plant leaves compared with wild type [122]. The expression of the ABA catabolic gene *CYP707A1* is increased by the application of synthetic strigolactone GR24, which decreased the ABA level and stimulated seed germination even under heat stress [123]. Moreover, by application of exogenous GR24, the ABA levels reduces in Arabidopsis seeds, thus reducing the dormancy induced by thermo-inhibition [124]. However, it was observed that GR24 increased GA4 levels in both wild-type and *max1*-*1* seeds at 32 °C, while *max 2*-*1* did not respond to GR24. Furthermore, the repression of a key enzyme in the production of bioactive GA, gibberellin 3-oxidase2 (GA3ox2), was significantly decreased at 32 °C compared to the levels at the optimal temperature, and treatment with GR24 suppressed the transcript abundance of 9-cis-epoxycarotenoid dioxygenase9 (NCED9), one of the key enzymes for ABA biosynthesis in seeds, similar to those seen at 24 °C [124]. Under normal or stress conditions, the ABA level does not change by SL-insensitivity [125]. Moreover, *MAX2*, the SLs receptor, plays a fundamental role in abiotic stress resistance at the shoot level, and that associates with the reduced response to exogenous ABA by guard cells [118,125]. Recently, a study revealed that SLs play a key role in activating *Slow Anion Channel Activating 1* (*SLAC1*) in inducing the closure of stomata in an ABA-independent manner (Figure 3) [126]. In addition, SLs function as a positive regulator in the defense against nematode attacks. ABA appears to act downstream of SL in the defense response to root-knot nematodes (RKNs) by suppressing the expression of *basic helix*–*loop*–*helix* (bHLH) transcription factor *MYC2*, which negatively regulates defense, whereas *Plant Defense Factor 1.2* (*PDF1.2*) and protein inhibitors (PI) play major roles in the SL-mediated defense responses (Figure 3) [127]. Exogenous ABA application may increase the accumulation of SLs during stressful situations [121]. A comparative analysis of SL mutants (*d3*, *d10*, *d17*, and *d27*) of *Oryza sativa* revealed that *d27* produced a low level of ABA as compared to other *dwarf* mutants, indicating that *D27* could be associated with the regulation of ABA [128]. Moreover, SLs improved the ability of stomatal movement as a systemic stress signal component in tomato (*Solanum lycopersicum*) [129]. Furthermore, both ABA and SLs are key regulators in salt stress response, as well as important for the symbiotic relationship between the host plant and AMF. SLs are thought to have key roles in adverse environments; however, these conclusions are mainly based on studies on Arabidopsis. Therefore, extended experiments with additional mutants may help to resolve the current ambiguities.

### 5.3. SL–Cytokinins Interaction: A Signal of Plant Nutritional Status

SLs and cytokinins are antagonistic hormones, however, both are produced in roots but communicate different messages to the shoot about the nutritional status. Generally, CKs communicate a positive message that promotes the growth of shoots, while negative information relies on SLs that generally inhibits the growth of shoots. Previous studies showed that SL mutants of pea have significantly decreased levels of CKs in the xylem [58]. Furthermore, the SL mutants of pea are oversensitive to CKs treatment, suggesting an antagonistic relationship between SLs and CKs. The decreased level of CKs in SL mutants of pea appears from the long-term developmental response, rather than from any effect on the synthesis of CKs by SLs’ signaling itself [130]. SLs significantly up-regulate the negative regulator of branching, i.e., *BRC1* (Figure 3) [131], Fine Culm1 (*FC1*) in rice [132], and *IPA1* [133]. Similarly, the hyper-sensitivity of SL mutants to CKs seems to arise from their antagonistic co-regulation of the Branched1 transcription factor in buds, rather than from any effect of SL on CKs signaling itself [131]. Notably, GR24 suppresses LR growth by down-regulating *PIN* gene expression, which is controlled by *AHK3*, *ARR1*, and *ARR12* via Short Hypocotyl2 (*SHY2*) (Figure 3). Because auxin transport is essential for the establishment of an auxin gradient, which is required to stimulate LR initiation from pericycle founder cells, blocking PIN activity with *SHY2* prevents the LR initiation [134]. Furthermore, CKs treatment has been shown to inhibit SL gene transcription in rice over a short time [132], but the phenotypic evidence is unclear. Recently, it was observed that the increasing amount of endogenous SLs in less-branched varieties may have repressed the concentration of endogenous CKs leading to the inhibition of axillary buds in the calla lily (*Zantedeschia aethiopica*) [135]. In general, CKs formation in the roots is mainly influenced by the nitrate levels in the soil, whereas phosphate levels primarily affect SL synthesis. This explains relatively less indication of cross-regulation between CKs and SLs. Even though they are directly opposite in function, both signals are needed for shoots to correctly measure growth responses to soil resource availability.

### 5.4. Antagonistic Interaction of SL with Gibberellic Acid (GA)

SLs and GAs intersect in plant development, such as leaf elongation and internode length. There is also a clear similarity between the SLs and GAs signaling mechanisms, with α/β hydrolase proteins (D14) and Gibberellin-Insensitive Dwarf1 protein (GID1) activating the degradation of target SMXL7/D53 and DELLAs proteins, respectively, in response to hormonal signals, mediated by SCF-type ubiquitin ligases [136]. Previous experiments proved that the degradation of DELLA proteins is targeted by SLs signaling, and GAs regulate SLs synthesis [38,137]. At present, these findings have not been confirmed, and there is little indication that supports the direct interaction of SLs with GAs. Moreover, there is very little phenotypic evidence between SLs and GA mutants, indicating that the hormones are generally regulated separately [138]. The GA inhibitor PAC (pacrobutrazol) inhibited the alleviation of heat inhibition by GR24, indicating that GA is necessary for SLs germination activity; additionally, GR24 can raise the endogenous GA4 level of seeds. GR24 also inhibited *NCED9* and *GA3ox2* expression, a bioactive enzyme in the production of GA, eventually resulting in the decrease of endogenous ABA and GA by optimizing the ABA:GA ratio in seeds imbibed at 32 °C (Figure 3) [124]. Previously, in stem elongation, genetic evidence shows a clear picture that SLs and GAs act autonomously [49,139]. The direct effects of SLs and GAs on transcription in seedlings are almost entirely additive, again suggesting that these hormones act independently. Contrarily, the SLs signaling also does not have any influence on the stability of DELLA proteins [138,139]. The long-term detrimental effects of GAs treatment on SLs biosynthesis gene expression and exudation of SLs from the root system in rice have been hypothesized [137]. However, this only occurs after 24 h of GA treatment and is not a direct response to GA treatment [137,139]. Overall, the functions of SLs and GAs in development are largely opposite, and in general, it would make little sense for them to interact in the manner previously proposed.

### 5.5. SLs and Salicylic Acid (SA) Regulatory Network

Drought tolerance of wheat plants was intensified in most of the cases when these phytohormones were used together, implying a possible interaction between salicylic acid (SA) and SL under drought situations [140]. Exogenous treatments of SA and SLs were recently found to abolish the damaging effects of drought in winter wheat plants [140]. Enhanced electrolyte leakage is a consequence of impaired membrane integrity, and it was shown in wheat under water stress condition [140]. Arabidopsis *max1* mutants accumulated considerably more SA than WT plants, whereas *max2* mutants acquired much less SA. The concentration of SA rose in WT and *max2* after inoculation, whereas *max1* mutants responded to inoculation with decreased SA accumulation considerably lower than in WT and *max2* [141]. SL deficiency altered defense-related hormone profiles: the *Slccd8* tomato RNAi line accumulated less JA and SA [122]. Exogenously applied synthetic SLs activated SA synthesis in *A. thaliana* but only in concentrations of 1 mM; lower concentrations did not affect SA production in seedlings [141]. SLs can link other hormone pathways and may form a regulatory network for various aspects of plant development and adaptation to abiotic stress. However, the interaction between SLs and other hormones under abiotic stress must be further explored using physiological, biochemical, genetic, and molecular biological methods.

### 5.6. SLs and Brassinosteroids (BRs) Signal Integration and Their Downstream Affects

A potential interaction between SLs and BR suggested previously that BES1, a transcription factor that mediates BR responses, is a proteolytic target of SL/SCFMAX2 signaling [142]. This notion is supported in part by enhanced branching in the original *bes1*-*d* mutant, in which BES1 is stabilized [142]. However, the re-analysis of a backcrossed *bes1* mutant line suggests that BES1 activity has no relevance for SL phenotypic effects and does not confer SL insensitivity [138]. Thus, although it cannot be ruled out that SL signaling (or indeed KL signaling) alters the stability of BES1, if this occurs, it seems to have little relevance for the well-defined effects of SLs on plant development. Notably, BRs have distinct roles in regulating rosette branching in Arabidopsis and tillering in rice, respectively. In Arabidopsis, BRs and their early signaling components upstream of AtBES1 do not regulate the primary branch number and *BRC1* expression (Wang et al., 2013); in rice, however, BRs and their early signaling significantly promote tillering by inhibiting *FC1* expression [143]. Genetic and biochemical evidence shows that the D53-OsBZR1 complex is required to integrate BR and SL signals to control rice tillering. The differential regulation of *AtBES1*/*OsBZR1* by BR signaling results in these diverse functions of BRs in Arabidopsis and rice [143].

### 5.7. Connections between SLs and Ethylene (ET) in Plant Biology

Leaf senescence is not only regulated by the developmental stage but in young plants is also influenced by numerous internal and environmental cues [84]. Plant hormones are internal signals for the development of plants and responses to biotic and abiotic stresses and play significant roles in leaf senescence. Several hormones such as ethylene, abscisic acid, jasmonic acid, and salicylic acid promote leaf senescence, whereas cytokinins are strong inhibitors of leaf senescence [85]. In addition, SLs, a new class of hormone, also regulate the senescence of leaves because biosynthetic and signaling mutants of SLs show delayed leaf senescence [18,87]. Recently, it was detected that the production of ethylene increased significantly by the treatment of GR24, while the effect of both an ethylene inhibitor (AVG) and GR24 in combination decreased ethylene production compared to GR24 treatment alone [144]. Furthermore, it was discovered that SLs and ethylene interact to modulate chlorophyll breakdown caused by darkness in perennial ryegrass [144].

It has also been proposed that SLs have a favorable influence on root hair (RH) elongation, which is mediated by the *MAX2*. Besides this, auxin and ethylene, two other phytohormones have been shown to have a significant function in RH elongation [83]. Nonetheless, the substantial response of the ethylene mutant *ein2*-*1* to GR24 in relation to RH elongation might propose that RH elongation by SLs is facilitated through both ethylene-dependent and -independent pathways. This residual response of ein2-1 to GR24 could be due to the result of SLs on RH elongation through the auxin response pathway (Figure 3) [83]. SL also promotes leaf senescence through the acceleration of chlorophyll degradation in concert with ethylene. The application of ethylene inhibitor 2-aminoethoxyvinyl glycine (AVG) maintains greener leaves compared to GR24 treatment by ameliorating the senescence trait caused by GR24 [144]. These findings suggested that the synergistic effects of SLs and ethylene may be persistent throughout plant growth. Previously it was concluded that, in the presence of an ethylene inhibitor 1-methylcyclopropene (1-MCP), CRT1 binds to the receptor, promoting the phosphorylation of EIN2, and therefore, blocking ethylene downstream signaling [145]. As such, there is a synergistic effect of SL on ethylene signaling, and it will be an interesting study to know the interaction among the SLs and ethylene under the application of ethylene inhibitor 1-MCP.

## 6. Altered SLs Metabolism through Gene Manipulation to Cope with Environmental Stresses

Agriculture is confronted with the tremendous challenges of ensuring long-term sustainability and feeding an expanding population in a changing climate and over the next three decades since the global population of humans is predicted to expand by 25%. As the occurrence of extreme weather increases due to climate change, developing crops tolerant to adverse conditions will become increasingly relevant globally. Crop varieties with modified SL production and signaling can be developed to manipulate SL signaling for agricultural gains. Striga species infest maize (*Zea maize*), rice (*Oryza sativa*), sorghum (*Panicum miliaceum*), and millet (*Urochloa ramosa*) crops across Africa, lowering maize yields up to 80% in heavily infested fields and generating significant loss and food insecurity [8].

Striga and/or Orobanche resistance can be seen in crops with reduced or impaired SL exudation and cultivars of rice (NERICA 1 and CG14), tomato (*Solanum lycopersicum*) (SL-ORT1), faba bean (*Vicia faba*) (G5), and pea (*Pisum sativum*) (ROR12) are among the crops that have developed resistant variants [146,147,148]. Sorgomol exudation was increased in striga-resistant maize (KST 94) against the susceptible maize, while similar levels of mycorrhization were recorded in both cultivars [149]. In addition, exogenous SLs improve drought tolerance in a variety of commercially produced crops [140,150]. Furthermore, by combining a changed root system architecture with faster mycorrhization, up-regulation of the SL transporter (*PDR1*) increased petunia (*Petunia hybrida*) phosphate intake and plant biomass [151]. In addition, semi-dwarfing rice (*Oryza sativa*) and wheat (*Triticum aestivum*) cultivars were significant boosters of the green revolution, which resulted in incredible gains in crop yield [152]. CRISPR/ Cas9-mediated editing of the SLs biosynthetic gene *CCD7* boosted tillering in the Nipponbare background, and altered levels of tillering among commercial rice cultivars correlate negatively with SL levels [153]. Likewise, improved grain yield in other cereal crops could benefit from genetic techniques to lower SL biosynthesis or perception. Furthermore, genetically manipulating SL signaling will necessitate significant progress in our understanding of SL synthesis pathways. Secondary growth, or the thickening of stems and roots as a result of cambium proliferation, is an essential agronomic feature that affects wood production and stem strength.

In Arabidopsis and pea, SL mutants were discovered to have thinner stems [154]. Auxin, which similarly increases cambium proliferation, appears to be downstream of the SLs’ activity in secondary growth [154]. Primary root length, lateral root development, and adventitious rooting are all thought to be regulated by SLs; however, because of the interaction with other phytohormones, the nature of these actions differs across species and growth conditions. The SL-signaling mutant *hvd14* in barley (*Hordeum vulgare*) showed shorter seminal roots, whereas GR24 application increased seminal roots in the wild type but not in the signaling mutant [155]; however, the role appears to be species specific. GR24 treatment increased root hair length in wild-type and SL-deficient Arabidopsis plants, but not in an SL-insensitive mutant [83]. Notably, auxin and ethylene both induce root hair elongation, and SLs may function upstream of the ethylene signaling pathway [83]. In addition, adventitious rooting was enhanced in SLs-signaling mutants of Arabidopsis and ipecac (*Carapichea ipecacuanha*), implying that SLs inhibit adventitious rooting [81,156].

SLs have been associated with enhanced drought tolerance and stomatal closure in a variety of taxa and appear to be essential for ABA response [71]. In Arabidopsis leaves, tomato shoots, and rice stem base, water deprivation elevated the expression of SL-biosynthesis genes [118,157]. SLs play a crucial function in nitrogen uptake in legumes by favorably regulating nodulation. In alfalfa (*Medicago sativa*), pea, and soybean (*Glycine max*) plants, GR24 increased nodulation, and SL-biosynthesis mutants had fewer nodules than wild-type plants in *L. japonicus*, pea, and soybean (*Glycine max*) [39,40,42]. This may lead to applications enhancing nutrient access and yield in leguminous crops.

Aside from their roles in plant architecture, SLs have also been identified as a key player in pathogen resistance [155]. The discovery of pathogen-associated TF motifs in the promoters of genes involved in SL biosynthesis was the first piece of evidence [122]. Arabidopsis *max2* and tomato *Slccd8* mutants are more susceptible to fungal pathogens *Botrytis cinerea* and *Alternaria alternata* [122,158]. Recently, it was discovered that SLs inhibit the *MYC2* expression and play a significant role in the tomato plant, possibly through interaction with ABA [127]. These diverse roles of SLs in plant biology make SL signaling a promising target for controlling parasitic weeds, optimizing crop architecture, enhancing nutrient acquisition, and increasing resilience to abiotic and biotic stresses. The above-mentioned examples demonstrate the influence of manipulating SL signaling; however, numerous SL activities are yet to be explored and the potential application of SLs remains to be tested.

## 7. Conclusions and Future Perspectives

Strigolactones are carotenoid-derived plant secondary metabolite molecules and act on different aspects of plants to regulate their development and growth, including seed germination, shoot branching, leaf senescence, root development, etc. Stress may have an impact on SL biosynthesis, signaling, and crosstalk with other plant hormones. In recent years, SLs have raised tremendous attention because of their significant roles in the adaptation of plants to several abiotic stresses, such as drought, salinity, nutrient deficiency, heat, chilling and heavy metals, as well as in control of a number of physiological and molecular processes.

Collectively, all of these areas signify the enormous impact of SLs in modern agriculture. However, many important questions remain to be answered in the future. With respect to SL biosynthesis, several hints have recently been reported but the bioactive form of SLs that regulates various aspects of plant growth and development is still uncertain. Further biochemical and genetic analysis of SL precursors and the identification of new enzymes will be key to understand the whole picture of the SL mechanism. Although there are many gaps in the transport and perception of SL in plants as PDR-mediated transport of SL is limited to some species, exploring the molecular mechanism underlying SL transport in other plant species will provide hints to understand the effective utilization of SL molecules. Additionally, the identification of the SLs receptor D14 in AMF will be an effective approach to identify the versatile function of SL. Moreover, exploring the intercellular transport of SLs’ precursors will increase our knowledge about the exact mechanism of SLs in planta.

Many studies have evidence that SL has multiple roles in the development of plants, but the pivotal role of other phytohormones’ action in enabling plants to cope with limiting and stressful ambient conditions cannot be overemphasized. The crosstalk of SLs with phytohormones provided further insights with regard to changes in the molecular structures, physiological processes, and biochemical mechanisms in plants. Besides, with the emerging roles of SLs in this aspect of plant development, it is imperative to characterize the crosstalk between SLs and these hormones to develop a model of the circuit of hormonal actions that drive plants’ innate response and defense mechanisms against biotic and abiotic stress in their environment. Additionally, the application of hormone inhibitors will increase our understanding and the underlying mechanism on the crosstalk of SLs with other phytohormones. Determining the biosynthetic processes involved in SLs and increasing the use of varied SLs in biological investigations will be critical for the development of modified agricultural cultivars that exploit SLs’ specificity. Moreover, the identification of genetic variation and favorable alleles of genes involved in SLs’ diversification and downstream signaling processes would be a valuable asset to future breeding operations, as it would aid in fine-tuning SLs’ function to maximize agricultural production.

## Figures and Tables

**Table 1:** Genes involved in strigolactones (SLs) biosynthesis and signaling.

Role	Protein Identity/ Function	Arabidopsis	Pea	Petunia	Rice	References
**SL biosynthesis**	9-cis/all-trans-β-Carotene isomerase	*AtD27*	-	-	*D27*	[3,4,65]
	Carotenoid cleavage dioxygenase7 (CCD7)	*MAX3*	*RMS5*	*DAD3*	*HTD1*/*D17*	[3,15,20,66]
	Carotenoid cleavage dioxygenase8 (CCD8)	*MAX4*	*RMS1*	*DAD1*	*D10*	[3,19,22]
	Cytochrome P450, cytochrome711 (CYP711)	*MAX1*	-	*PhMAX1*	Carlactone oxidase (Os01g0700900) Orobanchol synthase Os01g0701400 Os01g0701500 Os02g0221900 Os06g0565100	[15,20,67]
**SL perception**/ **signaling**	α/β-Hydrolase	*AtD14*	-	*DAD2*	*D14*/*D88*/*HTD2*	[2,18,38]
	F-box protein	*MAX2*	*RMS4*	*PhMAX2A PhMAX2B*	*D3*	[12,17,20,25]
	Class I Clp ATPase protein	*SMXL6*, *7*, & *8*	-	-	*D53*	[24,53]
	TCP transcription factor	*BRC1*	*PsBRC1*	-	*FC1*/*OsTB1*	[68]
**SL transport**	Pleiotropic Drug Resistance 1 (PDR1)			*PhPDR1*		[60,61]

## Data Availability

The data presented in this study are available in review.
